# Females and Males Contribute in Opposite Ways to the Evolution of Gene Order in *Drosophila*

**DOI:** 10.1371/journal.pone.0064491

**Published:** 2013-05-16

**Authors:** Carlos Díaz-Castillo

**Affiliations:** Irvine, California, United States of America; Spanish National Research Council (CSIC), Spain

## Abstract

An intriguing association between the spatial layout of chromosomes within nuclei and the evolution of chromosome gene order was recently uncovered. Chromosome regions with conserved gene order in the Drosophila genus are larger if they interact with the inner side of the nuclear envelope in D. melanogaster somatic cells. This observation opens a new door to understand the evolution of chromosomes in the light of the dynamics of the spatial layout of chromosomes and the way double-strand breaks are repaired in D. melanogaster germ lines. Chromosome regions at the nuclear periphery in somatic cell nuclei relocate to more internal locations of male germ line cell nuclei, which might prefer a gene order-preserving mechanism to repair double-strand breaks. Conversely, chromosome regions at the nuclear periphery in somatic cells keep their location in female germ line cell nuclei, which might be inaccessible for cellular machinery that causes gene order-disrupting chromosome rearrangements. Thus, the gene order stability for genome regions at the periphery of somatic cell nuclei might result from the active repair of double-strand breaks using conservative mechanisms in male germ line cells, and the passive inaccessibility for gene order-disrupting factors at the periphery of nuclei of female germ line cells. In the present article, I find evidence consistent with a DNA break repair-based differential contribution of both D. melanogaster germ lines to the stability/disruption of gene order. The importance of germ line differences for the layout of chromosomes and DNA break repair strategies with regard to other genomic patterns is briefly discussed.

## Introduction

The distinction between chromosome domains at the periphery and more internal locations of nuclei is a general characteristic of eukaryotes [1]–[6]. The peripheral component of the genome has been shown to differ from the rest of the genome in features such as nucleotide composition, gene density, chromatin structure, replication timing, gene expression, and damage repair [1]–[4], [7]–[16]. Thus, we can define the peripheral syndrome as the ensemble of features characteristic of the peripherome, the component of the genome occupying the nuclear periphery. The fraction of the genome that interacts with the nuclear lamina at the internal side of the nuclear envelope of *Drosophila melanogaster* somatic cells, hereinafter the *D. melanogaster* peripherome, has been shown to abound in chromosome domains with remarkable gene order stability in the *Drosophila* genus [17], [18]. Some of the features that characterize peripheromes, such as reduced gene expression, have been explained by the difficulty the corresponding cellular machinery has in accessing the nuclear periphery [19]. Therefore, a possible cause for the *D. melanogaster* peripherome gene order stability would be the limited accessibility of loci located at the nuclear periphery for cellular elements that cause gene order disrupting chromosome rearrangements. Since the disruption of gene order ultimately depends on DNA breaks, the cellular elements that cause gene order-disrupting chromosome rearrangements would include those that cause and repair DNA breaks.

The component of *D. melanogaster* genome that occupies the putatively inaccessible nuclear periphery in somatic and female germ line cells becomes accessible in male germ line cell nuclei, which might represent a hazardous environment for its integrity. Clusters of genes specifically expressed in testis that interact with the nuclear lamina in *D. melanogaster* somatic cells relocate to more internal locations of spermatocyte nuclei [20]. Also, the *D. melanogaster* peripherome is significantly enriched in genes with testis-biased expression and depleted in genes with ovary-biased expression, when compared with the rest of the genome [21]. Spermiogenesis, which consists of the postmeiotic maturation of spermatids into spermatozoa, is characterized by an almost complete replacement of histones by protamine or protamine-like proteins, leading to the high degree of chromatin compaction typical of sperm [22]–[25]. The histone-protamine replacement is facilitated by the formation of abundant DNA breaks that contribute to chromatin relaxation [22]–[24]. Therefore, the *D. melanogaster* peripherome gene order stability cannot be exclusively explained as a byproduct of its limited accessibility at the nuclear periphery.

Nevertheless, the *D. melanogaster* peripherome accessibility in male germ line cells points in a new direction to understand its gene order stability. The success rate of targeted mutagenesis strategies that take advantage of different double-strand break (DSB) repair mechanisms suggests that *D. melanogaster* female and male germ lines handle the repair of DSBs in different ways. Targeted mutagenesis that relies on the use of the premeiotic homologous recombination (HR) machinery to repair induced DSBs is considerably more efficient in females than in males [26]–[30]. Furthermore, HR-dependent targeted mutagenesis assayed in meiotic/postmeiotic stages of spermatogenesis resulted unsuccessful [31]. On the other hand, no consistent differences between females and males have been observed for targeted mutagenesis that relies on the use of the premeiotic non-homologous end-joining (NHEJ) machinery to repair induced DSBs [29]. Also, DSBs induced in meiotic/postmeiotic stages of spermatogenesis were preferentially repaired using NHEJ [32]. Thus, the *D. melanogaster* male germ line seems inefficient at repairing DSBs using HR mechanisms, but as efficient as the female germ line using NHEJ mechanisms. NHEJ preference seems to be especially accentuated in meiotic/postmeiotic stages of spermatogenesis, when the peripherome moves out of the nuclear periphery to more accessible regions.

The comparison of HR and NHEJ dynamics in the same cell system showed that the latter is considerably faster and more efficient than the former [33]. It has been suggested that faster and efficient DNA repair pathways will tend to rejoin the ends of each DSB [34], which might not allow for enough time for the broken DNA ends to invade loci with enough sequence complementarity, and prime the formation of cross-overs or gene order-disrupting chromosome rearrangements. Thus, it could be argued that the HR-inefficient NHEJ-efficient *D. melanogaster* male germ line could produce fewer meiotic recombination events and gene order-disrupting chromosome rearrangements. Interestingly, *D. melanogaster* males lack meiotic recombination, as most of Dipteran males do [35], [36]. Furthermore, a negative correlation has been found between *D. melanogaster* meiotic recombination rates and the length of gene order conservation in the genus *Drosophila* [18], [37]. On the other hand, the possible effects of the putative NHEJ-bias in the male germ line suggests that most of gene order disruption occurs, along with recombination, in the *D. melanogaster* female germ line, which prefers HR to NHEJ for the repair of DSBs [29]. Indeed, a positive correlation has been found between *D. melanogaster* meiotic recombination rates and the number of gene order-disrupting chromosome rearrangement breakpoints in the *Drosophila* genus [37].

Therefore, *D. melanogaster* germ lines might contribute in completely different ways to the remarkable gene order stability of the *D. melanogaster* peripherome. On one hand, the preferential usage of NHEJ to repair DSBs in the peripherome, because of its relocation to more internal locations in meiotic/postmeiotic male germ line cell nuclei, would result in the active preservation of its gene order. On the other hand, the inaccessibility of the peripherome in female germ line cell nuclei for cellular elements that can promote gene order-disrupting chromosome rearrangements, such as the HR machinery, would result in the passive preservation of its gene order.

In the present article, I test whether *Drosophila* germ lines contribute in different ways to the preservation/disruption of gene order by answering three questions. Does the *D. melanogaster* peripherome show evidence of preferential NHEJ usage to repair DSBs, compatible with its accessibility in the putatively NHEJ-biased male germ line? Does gene order disruption occur more often in the *Drosophila* female germ line, compatible with the lower production of gene order-disrupting chromosome rearrangements in the putatively NHEJ-biased male germ line? Finally, do *Drosophila* germ lines contribute in different ways to the evolution of gene order?

## Results

### Does the D. melanogaster peripherome show evidence of preferential NHEJ repair?

NHEJ is a remarkably flexible process, not just because it acts upon very diverse disrupted DNA ends, but also because the succession of participating enzymes, including nucleases, polymerases, and ligases, can proceed differently even for two joined DNA ends [38]. Such flexibility is manifested in the accentuated heterogeneity at NHEJ-repaired loci [38]. Although the diversity of NHEJ-repaired products is a common theme to all species there are clear differences in the range of these products between distantly related species [38], [39]. Thus, when looking for traces of NHEJ it is better to rely on information of the species under study over better known, but distantly related, species. The study of NHEJ products in *D. melanogaster* shows that insertions and deletions occur frequently [29], [30], [40]–[46]. The insertion of genetic elements that move across the genome using RNA-intermediates, such as RNA-mediated duplicated genes or retrogenes, takes advantage of NHEJ machinery [47]. Recently, an excess of retrogenes putatively originated in the *Drosophila* male germ line was found in the *D. melanogaster* peripherome, consistent with its accessibility in a moment where there might be a preference to use NHEJ to repair DSBs [21]. This trend was uncovered after studying 69 retrogenes originated over the last 63 million years of the evolution of the Drosophila genus [21], [48], constituting a dataset with a very limited number of events occurred over a long evolutionary time.

**Figure 1:**
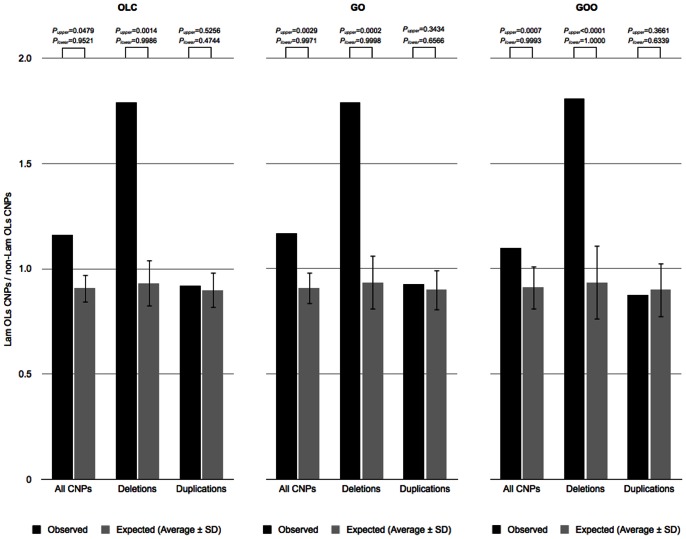
OL distribution of D. melanogaster CNPs. OL distribution of CNPs was studied according to three reconstructions of *Drosophila* genus gene order evolution: OLC, GO, and GOO (see main text for definitions). Lam OLs were used as proxy for the *D. melanogaster* peripherome. Expected measures were obtained after assigning new random locations to all CNPs, respecting their chromosome arm distribution (10,000 replicates). *P*_upper_ and *P*_lower_ values represent the fraction of random simulations with measures larger or equal, and lower or equal than the observed ones, respectively.

If the preference to repair DSBs by NHEJ in the peripherome in the male germ line truly exists, we should expect to see clear signs of NHEJ-mediated DSB repairs in datasets of genetic variation occurred over shorter periods of time. Thus, I analyzed the genomic distribution of 2,658 independent copy-number polymorphisms (CNPs) from 15 *D. melanogaster* natural isofemale lines [49]. Since the association between gene order stability and NHEJ-biased repair is the focus of the analysis, I quantified the number of CNPs within those chromosome domains defined by their internal gene order stability in the *Drosophila* genus, or orthologous landmarks (OLs) [50]. The analysis was performed according to three definitions of gene order stability: OLC, when an overall local gene contiguity is conserved between *Drosophila* species, GO, when only gene order is conserved between *Drosophila* species, and GOO, when both gene order and orientation are conserved between *Drosophila* species [50]. As a proxy for the *D. melanogaster* peripherome, I used OLs that contain at least one gene interacting with the nuclear lamina or Lamin target gene (Lam OLs) [4], [50] (Dataset S1). To account for the distribution of CNPs caused by factors other than the accessibility in the putatively NHEJ-biased male germ line of the *D. melanogaster* peripherome, OLs containing no Lamin target genes (non-Lam OLs) were used as a reference in the analysis (Dataset S1). Regardless of the definition for gene order stability, a significant excess of CNPs was found within Lam OLs when compared with non-Lam OLs (Figure 1, and Table S1). The excess of CNPs in the peripherome is based, exclusively, in a significantly larger ratio of deletions between Lam OLs and non-Lam OLs (Figure 1, and Table S1).

The significant excess of *Drosophila* retrogene insertions [21], and *D. melanogaster* polymorphic deletions found at the *D. melanogaster* peripherome are consistent with the types of genetic variation that are expected after the NHEJ-preference to repair DSBs, likely due to its atypical accessibility in the male germ line. However, other factors could also account for these patterns. The contribution of HR pathways, such as single-strand annealing (SSA) or unequal sister chromatid exchange, to the excess of deletions in the peripherome is expected to be very limited, since the use of these pathways is far less common than NHEJ in *D. melanogaster* male germ line [32]. Also, in *D. melanogaster* a still not well understood association has been found between polymorphic deletion or duplication hotspots and early or late replication, respectively [51], [52]. One of the characteristics of the *D. melanogaster* Lamin target genes is that they replicate late [4]. Thus, we should expect to see for the peripherome an excess of duplications, but not deletions. Future analyses are required to clarify this inconsistency.

### Does gene order disruption occur more often in the Drosophila female germ line?

**Figure 2:**
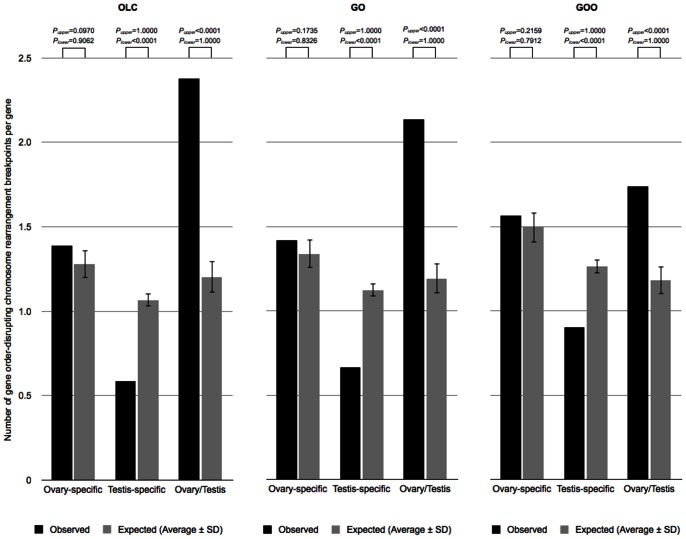
Association between gene order disruption and gonadal gene expression. The association of gene order-disrupting chromosome rearrangement breakpoints with ovary- and testis-specific genes was studied according to three reconstructions of *Drosophila* genus gene order evolution: OLC, GO, and GOO (see main text for definitions). Expected measures were obtained after random permutation of testis- and ovary-specific gene tags, respecting their chromosome arm distribution (10,000 replicates). *P*_upper_ and *P*_lower_ values represent the fraction of random simulations with measures larger or equal, and lower or equal than the observed ones, respectively.

The evidence consistent with the preferential usage of gene order-preserving NHEJ mechanism in the male germ line would imply that in *Drosophila* gene order-disrupting chromosome rearrangements are mostly of female origin. To test this hypothesis, I measured the number of gene order-disrupting chromosomal rearrangement breakpoints at either side of genes putatively accessible in the female and the male germ lines [50] (Dataset S2). I considered that a gene that is expressed in the germ line might be also accessible to nuclear elements other than the transcription machinery. In the absence of comparable datasets in other *Drosophila* species, I used the *D. melanogaster* gonadal expression of OL genes as proxy for germ line accessibility [53]. Ovary-specific genes, which are expressed in ovaries and not in testes in *D. melanogaster*, are considered to represent genes that are accessible to the active machinery of the female germ line. Testis-specific genes, which are expressed in testes and not in ovaries in *D. melanogaster*, are considered to represent genes that are accessible to the active machinery of the male germ line. Regardless of the gene order stability definition used to infer the distribution of gene order-disrupting chromosomal rearrangement breakpoints [50], the ratio of breakpoints per gene between ovary- and testis-specific genes is larger than expected by chance (Figure 2). This excess is based on a significantly lower number of breakpoints per gene for testis-specific genes (Figure 2). These results are consistent with a net production of gene order-disrupting chromosome rearrangements in the female germ line, ultimately caused by a lower incidence of such changes in the putatively NHEJ-biased male germ line.

### Do Drosophila germ lines contribute in different ways to the evolution of gene order?

**Figure 3:**
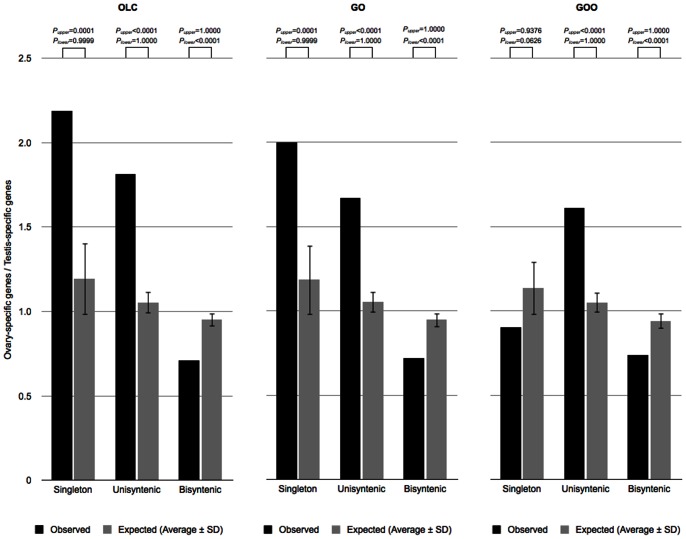
Association between gonadal gene expression and the evolution of gene order. Singleton and unisyntenic genes represent genes where gene order was disrupted at one or both sides in the *Drosophila* genus, respectively. Bisyntenic genes represent genes with complete gene order stability in the *Drosophila* genus (see main text for detailed definitions). The association between gene order stability/disruption and gonadal specific gene expression was studied according to three reconstructions of *Drosophila* genus gene order evolution: OLC, GO, and GOO (see main text for definitions). Expected measures were obtained after random permutation of testis- and ovary-specific gene tags, respecting their chromosome arm distribution (10,000 replicates). *P*_upper_ and *P*_lower_ values represent the fraction of random simulations with measures larger or equal, and lower or equal than the observed ones, respectively.

The preceding results suggest that *Drosophila* females and males contribute differently to the gene order preservation/disruption of chromosome domains specifically accessible in each germ line. The putative NHEJ-preference in the male germ line would result in the active preservation of gene order of chromosome domains accessible in the male germ line, whereas the putative net production of gene order disruptions in the female germ line would mainly affect genes accessible in the female germ line. To further test whether both germ lines differentially contribute to the evolution of gene order in the *Drosophila* genus, I estimated the ratio of ovary- and testis-specific genes in three groups of genes defined by changes in their flanking genes in the *Drosophila* genus [50], [53] (Dataset S2). A gene was deemed *singleton* if each of its flanking genes changed at least once. A gene was deemed *unisyntenic* if one of its flanking genes changed at least once, but the other one never changed. Finally, a gene was deemed *bisyntenic* if both flanking genes never changed. The proportion of ovary- and testis-specific genes is significantly different across classes of OL genes (Table 1). Furthermore, classes of genes with changes in at least one flanking gene, namely singletons and unisyntenic genes, show a significant excess of female-specific genes, whereas bisyntenic genes, with no changes at either side, show a significant excess of testis-specific genes (Figure 3, and Table S3). These results are consistent with the expected contribution of each germ line to the preservation/disruption of *Drosophila* gene order.

**Table 1:** Distribution of ovary- and testis-specific genes within three classes of OL genes according to the changes in their flanking genes in the *Drosophila* genus.

		OLC ^1^	GO ^1^	GOO ^1^
Singletons ^2^	Ovary-specific ^3^	32	35	48
	Testis-specific^ 3^	47	56	166
Unisyntenic genes^ 2^	Ovary-specific^ 3^	230	234	252
	Testis-specific ^3^	408	449	488
Bisyntenic genes ^2^	Ovary-specific ^3^	295	286	251
	Testis-specific^ 3^	1336	1272	1063
Chi-square tests for trend	Chi-square	89.35	73.13	22.56
	Degrees of freedom	1	1	1
	*P* value	<0.0001	<0.0001	<0.0001

1 Gene order stability definitions [50]: OLC, overall local gene contiguity; GO, gene order; GOO, gene order and orientation.

2 Gene classes defined by the changes in their flanking genes in the Drosophila genus [50]: singleton, each of its flanking genes had changed at least once; unisyntenic gene, one of its flanking genes had changed at least once, but the other one never changed; bisyntenic gene, both flanking genes never changed.

3 Gonadal gene expression [53]: ovary-specific gene, at least one of its probes was deemed as “present” in more than two ovary hybridizations (out of four), and none of its probes was deemed as “present” in more than two testis hybridizations (out of four); testis-specific gene, at least one of its probes was deemed as “present” in more than two testis hybridizations (out of four), and was not deemed as “present” in more than two ovary hybridizations (out of four).

It is interesting to note that the ratio of ovary- and testis-specific genes for singletons under GOO definition is not statistically significant, as it is the case for singletons under OLC, and GO definitions (Figure 3, and Table S3). This is consequence of the larger number of testis-specific genes found as singletons under GOO definition than under OLC and GO definitions (Table 1). The GOO definition requires the conservation of both gene order and orientation, so short inversions involving one or very few genes are interpreted as disruptions of gene collinearity [50]. These results would suggest that although chromosome rearrangements are also produced in the putatively NHEJ-biased male germ line, they are small and do not disrupt the general collinearity of larger blocks of genes.

## Discussion

The limited amount of systematic information at genomic and molecular levels for *Drosophila* species other than *D. melanogaster* means that in many cases we had to rely on the information from a single species to test hypotheses for the evolution of genomes at the genus level. This is the case for some of the features analyzed or referred to in the present article, such as the interaction with the nuclear lamina, recombination rates, replication timing, or gene expression. This poses a problem since these features could diverge considerably between species, so such studies need to be interpreted with caution until more information is available. Because of its transcendence in the present article, the case of the gonadal gene expression is particularly noteworthy. It is known that gonadal gene expression, especially in testis, changes considerably in the *Drosophila* genus [54], [55]. A way to overcome this problem would be to restrict the analyses to species closely related to *D. melanogaster*, *i.e.* the *D. melanogaster* species subgroup. However, such approach comes with a considerable reduction of analytical power, since the number of chromosome disruptions estimated for the *D. melanogaster* species subgroup represents only ∼1% of the disruptions for whole genus [50]. Beyond phylogenetic conservation, there are two other caveats for the use of gonadal expression as a proxy for the accessibility of genes in the germ line cell nuclei. First, the gonads also consist of somatic cells, so, within the set of genes considered specifically expressed in the germ lines there might be genes that are expressed only in somatic cells of the gonads. Second, the gonadal gene expression dataset employed here might not represent adequately any temporal changes along gametogenesis [53], so, the set of genes used in the present analyses might include some that are not expressed/accessible when DSBs are produced and repaired. In principle, the use of datasets for gene expression in non-gonadal tissues [53], sex biased expression in other *Drosophila* species [55], or temporal changes in expression along spermatogenesis [56] could help refine the analyses performed here to focus on genes that are expressed/accessible when more accentuated is the difference for germ line DSB repair. However, none of these refinements are exempt of their own caveats, and could remove pertinent genes from the datasets under study.

The choice of OLs as base for the CNP-based search of NHEJ traces in the *D. melanogaster* peripherome could also be contested. Lam OLs can be considerably long (Table S4) [18]. The effect of heterogeneity in nuclear dynamics within each Lam OL on these results remains to be tested. The use of *D. melanogaster* testis-specific gene clusters, which tend to be located in the nuclear periphery of somatic cells [20], as the base for the CNP analysis could be interesting as complement to OL-based analyses. However, on average, these clusters are very small (Table S4) [20]. Gene cluster-based analyses could be more sensitive to distortions resulting from selective constraints on genetic elements contained in the clusters, and possibly harder to interpret. OL-based analyses, although not perfect, have the advantage of being potentially less distorted by gene-based selective constraints. Future experimental strategies resulting in genomic maps of gene expression-independent germ line accessibility, together with a better understanding of the way germ lines deal with DSBs in different *Drosophila* species will be of great importance to validate and expand the conclusions I reached herein.

It would be interesting to study how the differences between germ lines relate to other characteristics of the peripherome, or other factors constraining the evolution of gene order. For instance, genes with testis-biased expression in *D. melanogaster* are significantly enriched in the peripherome [21], and Lamin target genes are known to be located in chromosome regions with low gene density [4]. Thus, it could be inferred that the *D. melanogaster* peripherome had large intergenic distances and low gene density. The negative correlation between intergenic distance and gene order conservation found in yeast suggests that genes separated by short distances are less susceptible to be separated by chromosome rearrangements [57]. A similar trend in *Drosophila* would contrast with the results presented here. However, it seems that gene pairs whose collinearity was disrupted in the *Drosophila* genus have shorter intergenic distances in *D. melanogaster* [37]. This trend seems to fit better with the possibility that chromosome regions with low gene density, such as the peripherome, are less accessible in the female germ line, where most of gross chromosomal rearrangements might be produced. Whether there is a causal association between accessibility in the putative NHEJ-biased male germ line and low gene density, or other features that distinguish the peripherome from the rest of the genome, remains unclear.

How does germ line DSB repair relate with other constraints for the evolution of gene order? The two main factors considered to constrain the evolution of gene order are the non-homogenous distribution of genetic elements that participate in chromosome rearrangements, and the range and the non-homogenous distribution of regulatory interplays between genetic elements whose disruption is detrimental [50], [58]. On one hand, non-random distributions of genomic elements that can participate in these rearrangements, and/or confer fragility, would result in the empirically demonstrated reuse of breakpoints, namely the concentration of gene order-disrupting chromosome rearrangement breakpoints, and the preservation of gene order wherever these genomic elements are missing [59]–[65]. On the other hand, the common existence of clusters of genes that are coexpressed, expressed in a spatiotemporally concerted fashion, are related by other functional classifications such as Gene Ontology, or the association between gene interdigitation and long-range *cis*-regulators of gene expression with regions of gene order stability, have been interpreted to result from the detrimental effect the disruption of such ensembles would have [66]–[74]. It has been suggested that the non-random evolution of chromosome gene order would be under selective constraint derived from these or other type of functional interactions. These two models are not mutually exclusive [50].

The possibility that gene order evolution is influenced by the accessibility in germ lines with different DSBs repair preference would be hard to separate from the effect of the non-random distribution of genomic elements that can participate in chromosome rearrangements. Both would result in non-homogenous distributions of gene order-disrupting chromosome rearrangement breakpoints. On the other hand, a consequence of the NHEJ-dependent decreased probability for gene order disruption of certain chromosome regions would be an increased chance to establish long lasting regulatory interplays between the genetic elements within. Such derived regulatory interplays could subsequently contribute to the stability of these chromosome regions if their disruption became detrimental for the carriers. In other words, regulatory constraints on gene order disruption might ultimately be derived from the preexisting gene order stability provided by mechanisms such as NHEJ. Conversely, not all regulatory interplays found in evolutionary stable chromosome regions would actually contribute to the stability of these regions.

Highly conserved non-coding DNA elements (HCNEs) putatively act as long-range *cis*-regulators of gene transcription [73]. The over-representation of HCNEs in evolutionary stable chromosome regions in vertebrates and *Drosophila* species drove the hypothesis that HCNEs have an important role as regulatory constraints for the evolution of gene order [50], [73], [75]. However, the empirical disruption of a *D. melanogaster* group of genes with putative HCNE-dependent gene order stability did not result in the expected detrimental effect [76]. The chromosome region in question did not lack the kind of genetic elements commonly associated with chromosome rearrangements [76]. Thus, for this particular region, neither the presence of functional interplays, nor the absence of genomic elements that might participate in chromosome rearrangements, completely explain its remarkable gene order stability in the *Drosophila* genus. Intriguingly, this region could embody the different germ line contribution to the *D. melanogaster* peripherome gene order stability. This region shows clear evidence of being located in the nuclear periphery in somatic cells, and includes abundant genes expressed in testes but not in ovaries in *D. melanogaster* [76]. Since the *D. melanogaster* peripherome shows a larger fraction of HCNEs than the rest of the genome [18], [77], HCNEs could be one type of interplay arising in already-stable chromosome regions, of which only some will end up contributing to these regions stability. The possibility that NHEJ-based gene order stability promoted the onset of regulatory constrains for gene order evolution could imply that taxa with different usage of NHEJ to repair DNA breaks in germ cell nuclei would also show very different proportions of their genomes constrained by regulatory interplays. The integration of knowledge about the spatial layout of genomes, and the repair of DNA breaks in germ cell nuclei is of a great importance for better understanding the evolution of chromosomes.

Lastly, the study of taxa where germ line repair of DNA breaks differed drastically from *Drosophila* could be of great importance to evaluate the influence germ line biology has for the origin of genetic variation. One of such cases is Lepidoptera, whose females are the ones that are heterogametic (*ZW versus* the *ZZ* males), and lack meiotic recombination [78]. If the absence of meiotic recombination in Lepidopteran females is associated with an NHEJ-preference to repair DSBs, as it seems to be the case in *Drosophila* males, it should be possible to find NHEJ traces in chromosome regions that are accessible in the female germ line of Lepidoptera. Interestingly, a recent article showed that an excess of Lepidoptera retrogenes had originated from genes in the *Z* chromosome, but inserted into autosomes [79]. As opposed to taxa with heterogametic males, such as *Drosophila*, the expression of Lepidoptera retrogenes tends to be ovary-biased [79]. The study of the germ line accessibility of chromosome regions where Lepidopteran retrogenes landed will help calibrating the importance of germ line nuclear dynamics in explaining retrogene genomic distributions [21]. On the other hand, if gene order-disrupting chromosome rearrangements occur more often in the HR-efficient Lepidopteran male germ line, as it might be the case for *Drosophila* females, it should be expected that gene order disruption were particularly noticeable for genes accessible in the Lepidopteran male germ line. Lepidopteran chromosomes are extremely dynamic, being very small the size of groups of genes with conserved gene order and orientation [80]. It has been proposed that such malleability might derive from the amount and distribution of transposable elements and the holocentric nature of Lepidopteran chromosomes [80], [81]. *Z* is the most dynamic Lepidopteran chromosome, both within and between species [81], [82]. Interestingly, *Z* might be remarkably accessible in the male germ line cell nuclei, since it is the chromosome that contains the largest number of testis-specific genes [83]. In summary, the contribution of *Drosophila* and Lepidoptera germ lines for the origin of genetic variation might entail more similarities than one would expect after their obvious differences.

## Conclusions

In the present study, I found evidence of a differential contribution of the *Drosophila* germ lines to the evolution of gene order. In opposition to other explanations for the non-random evolution of gene order that rely on the non-homogeneous distribution of genetic elements, I conclude that a factor extrinsic to genomes, such as the repair of DNA breaks in the germ line, might be key for the evolution of chromosomes. The present study shows the importance a deeper knowledge of the context where genetic variation occurs has to understand evolutionary biases at the genomic level.

## Materials and Methods

### Original datasets

The original source of the analyzed datasets are: OL coordinates, OL gene content, and number of gene order-disrupting chromosome aberrations breakpoints at either side of OL genes [50], Lamin target genes [4], *D. melanogaster* natural isofemale lines CNPs [49], and, *D. melanogaster* gonadal gene expression [53]. Names and coordinates of OL genes, Lamin target genes, and CNPs were updated according to release 5.48 of *D. melanogaster* genome assembly using FB2012_06 (released in November 2012) [84]. FlyAtlas *D. melanogaster* gonadal expression data was used to define germ line accessibility for OL genes [53]. A gene was considered specifically accessible in the female germ line if its expression was ovary-specific, namely at least one of its probes was deemed *present* in more than two ovary hybridizations (out of four), and none of its probes was deemed *present* in more than two testis hybridizations (out of four). A gene was considered specifically accessible in the male germ line if its expression was testis-specific, namely at least one of its probes was deemed as *present* in more than two testis hybridizations (out of four), and was not deemed as *present* in more than two ovary hybridizations (out of four).

### CNP analysis

The statistical significance of the ratio of Lam OLs CNPs and non-Lam OLs CNPs was evaluated by performing Monte Carlo simulations (Figure 1, and Table S1). All CNPs were assigned random locations respecting their chromosome arm assortment (10,000 replicates). To account for the effect the different chromosome distributions and lengths of Lam OLs and non-Lam OLs might have on the analysis, the ratio of Lam OLs CNPs and non-Lam OLs CNPs for observed and simulated CNP distributions was calculated as *{[∑ ((xi/Xi)/Ai)]/L}/{[∑ ((xj/Xj)/Aj)]/nL}*, where, *i* and *j* stand for Lam OLs and non-Lam OLs, respectively, *x* represents the number of CNPs with at least one coordinate comprehended within the limits of each OL, *X* represents the number of CNPs in the chromosome arm each OL is located, *A* represents the length in bp for each OL, and, *L* and *nL* represents the number of Lam OLs and non-Lam OLs, respectively. 83%, 82%, and 78% of all CNPs were found within OLs according to OLC, GO, and GOO definitions of gene order stability [50], respectively. 78%, 76%, and 71% of deletions were found within OLs according to OLC, GO, and GOO definitions of gene order stability [50], respectively. 86%, 85%, and 82% of duplications were found within OLs according to OLC, GO, and GOO definitions of gene order stability [50], respectively.

### Gene order-disrupting chromosome rearrangement breakpoints analysis

The number of gene order-disrupting chromosome rearrangement breakpoints per gene was calculated by adding the number of breakpoints at either side of the gene in question [50]. The statistical significance of the number of breakpoints per gene for ovary- and testis-specific genes was evaluated by performing Monte Carlo simulations (Figure 2, and Table S2). Gonadal expression OL gene tags were randomly rearranged respecting their chromosome arm assortment (10,000 replicates). The ratio of breakpoints per gene for ovary- and testis-specific genes for observed and random distributions of gonadal gene expression tags were calculated as *(No/O)/(Nt/T)*, where *No* and *Nt* represent the number of breakpoints at either side of ovary- and testis-specific genes, respectively, and, *O* and *T* represent the number of ovary- and testis-specific genes, respectively.

### Association between gonadal gene expression and gene order stability/disruption in the Drosophila genus

OL genes were divided into three groups according to the changes in their flanking genes in the *Drosophila* genus [50]. An OL gene was deemed *singleton* if each of its flanking genes changed at least once in the *Drosophila* genus. An OL gene was deemed *unisyntenic* if one of its flanking genes changed at least once, but the other one never changed in the *Drosophila* genus. An OL gene was deemed *bisyntenic* if its flanking genes never changed in the *Drosophila* genus. Nested genes were always deemed *bisyntenic*. Genes hosting nested genes and changes for flanking genes at either side were deemed *unisyntenic*. The statistical significance of the proportion of ovary- and testis-specific genes across OL gene groups was estimated by performing Chi-square tests for trend (Table 1). The statistical significance of the ratio of ovary- and testis-specific genes for each class was evaluated by performing Monte Carlo simulations (Figure 3, and Table S1). Gonadal expression OL gene tags were randomly rearranged respecting their chromosome arm assortment (10,000 replicates). The ratio of ovary- and testis-specific genes for each class of OL genes in observed and random distributions of gonadal gene expression tags were calculated as *(no/No)/(nt/Nt)*, where *no* and *nt* represent the number of ovary- and testis-specific genes in each class of genes, respectively, and, *No* and *Nt* represent the number of ovary- and testis-specific genes in all three classes of genes, respectively.

### Statistics

All Monte Carlo simulations were performed using Microsoft® Excel® for Mac 2011 (Microsoft Corporation). *P*_upper_ and *P*_lower_ were calculated as the fraction of random simulations with larger or equal, and smaller or equal measures than the observed ones, respectively. Chi-square tests for trend were performed using Prism 5 for Mac OS X (GraphPad Software, Inc).

## Supporting Information

Table S1 **Monte Carlo simulations results: CNPs.**
(PDF)

Table S2 **Monte Carlo simulations results: gene order-disrupting chromosome rearrangement breakpoints per ovary- and testis-specific genes.**
(PDF)

Table S3 **Monte Carlo simulations results: ovary- and testis-specific genes in OL gene classes depending on the changes in their flanking genes in the Drosophila genus.**
(PDF)

Table S4 **Size of Drosophila Lam OLs and D. melanogaster testis-specific gene clusters.**
(PDF)

Dataset S1 **Number of CNPs per OL.**
(XLSX)

Dataset S2 **Number of gene order-disrupting chromosome rearrangement breakpoints, classification according to changes in their flanking genes, and D. melanogaster gonadal expression for Drosophila OL genes.**
(XLSX)
