# Supplementary material for: Females and Males Contribute in Opposite Ways to the Evolution of Gene Order in Drosophila
Source: PLoS One. 2013 May 16;8(5):e64491. doi: 10.1371/journal.pone.0064491 (PMC3655977; doi:10.1371/journal.pone.0064491)
Supplement: Table S1 — Monte Carlo simulations results: CNPs. (PDF) [file pone.0064491.s001.pdf]

Table S1. Monte Carlo simulations results: CNPs.

CNP type [1]	Gene order stability definition [2]	Observed [3]	Expected (Average ± SD) [3]	P_{upper} value [4]	P_{lower} value [4]
All	OLC	1.10	0.91 ± 0.104	0.0479	0.9521
All	GO	1.81	0.93 ± 0.176	0.0014	0.9986
All	GOO	0.87	0.90 ± 0.130	0.5256	0.4744
Deletions	OLC	1.17	0.91 ± 0.077	0.0029	0.9971
Deletions	GO	1.79	0.93 ± 0.128	0.0002	0.9998
Deletions	GOO	0.93	0.90 ± 0.095	0.3434	0.6566
Duplications	OLC	1.16	0.91 ± 0.067	0.0007	0.9993
Duplications	GO	1.79	0.93 ± 0.111	<0.0001	1.0000
Duplications	GOO	0.92	0.90 ± 0.084	0.3661	0.6339

1 Original CNP dataset obtained from Emerson JJ, Cardoso-Moreira M, Morevitz JO, Long M (2008) *Science* 320:1629-1631.
2 Gene order stability definitions according to von Grotthuss M, Ashburner M, Ranz JM (2010) *Genome Res*. 20:1084-1096. OLC, overall gene contiguity; GO, gene order; GOO, gene order and orientation.
3 Ratio of CNPs in Lam OLs and non-Lam OLs calculated for observed and expected by chance distributions of CNPs as: $\{[\sum ((x_i / X_i) / A_i)] / L\} / \{[\sum ((x_j / X_j) / A_j)] / nL\}$, where, *i* and *j* stand for Lam OLs and non-Lam OLs, respectively, *x* represents the number of CNPs with at least one coordinate comprehended within the limits of each OL, *X* represents the number of CNPs in the chromosome arm each OL is located, *A* represents the length in bp for each OL, and, *L* and *nL* represents the number of Lam OLs and non-Lam OLs, respectively.
4 P_{upper} and P_{lower} values represent the fraction of random simulations with ratios larger or equal, and lower or equal than the observed ones, respectively.
