# Supplementary material for: Females and Males Contribute in Opposite Ways to the Evolution of Gene Order in Drosophila
Source: PLoS One. 2013 May 16;8(5):e64491. doi: 10.1371/journal.pone.0064491 (PMC3655977; doi:10.1371/journal.pone.0064491)
Supplement: Table S2 — Monte Carlo simulations results: gene order-disrupting chromosome rearrangement breakpoints per ovary- and testis-specific genes. (PDF) [file pone.0064491.s002.pdf]

Table S2. Monte Carlo simulations results: gene order-disrupting chromosome rearrangement breakpoints per ovary- and testis- genes.

Gene expression [1]	Gene order stability definition [2]	Observed [3]	Expected (Average ± SD) [3]	P_{upper} value [4]	P_{lower} value [4]
Ovary-specifc	OLC	1.39	1.28 ± 0.084	0.0970	0.9062
Ovary-specific	GO	0.58	1.07 ± 0.039	1.0000	<0.0001
Ovary-specific	GOO	2.38	1.20 ± 0.094	<0.0001	1.0000
Testis-specific	OLC	1.42	1.34 ± 0.085	0.1735	0.8326
Testis-specific	GO	0.66	1.12 ± 0.040	1.0000	<0.0001
Testis-specific	GOO	2.14	1.19 ± 0.091	<0.0001	1.0000
Ovary/Testis	OLC	1.57	1.50 ± 0.089	0.2159	0.7912
Ovary/Testis	GO	0.90	1.27 ± 0.042	1.0000	<0.0001
Ovary/Testis	GOO	1.74	1.18 ± 0.084	<0.0001	1.0000

1 Original gene expression dataset from Chintapali VR, Wang J, Dow JA (2207) *Nat. Genet.* 39:715-720. Ovary-specific gene, at least one of its probes was deemed as "present" in more than two ovary hybridizations (out of four), and none of its probes was deemed as "present" in more than two testis hybridizations (out of four); testis-specific gene, at least one of its probes was deemed as "present" in more than two testis hybridizations (out of four), and was not deemed as "present" in more than two ovary hybridizations (out of four).
2 Gene order stability definitions according to von Grotthuss M, Ashburner M, Ranz JM (2010) *Genome Res*. 20:1084-1096. OLC, overall gene contiguity; GO, gene order; GOO, gene order and orientation.
3 Ratio of gene order-disrupting chromosome rearrangements breakpoints per gene in ovary- and testis-specific genes calculated for observed and expected by chance distributions of gonadal gene expression tags as: $(N_o / O) / (N_t / T)$, where N_o and N_t represent the number of breakpoints at either side of ovary- and testis-specific genes, respectively, and, O and T represent the number ovary- and testis-specific genes, respectively.
4 P_{upper} and P_{lower} values represent the fraction of random simulations with ratios larger or equal, and lower or equal than the observed ones, respectively.
