# Supplementary material for: Females and Males Contribute in Opposite Ways to the Evolution of Gene Order in Drosophila
Source: PLoS One. 2013 May 16;8(5):e64491. doi: 10.1371/journal.pone.0064491 (PMC3655977; doi:10.1371/journal.pone.0064491)
Supplement: Table S3 — Monte Carlo simulations results: ovary- and testis-specific genes in OL gene classes depending on the changes in their flanking genes in the Drosophila genus. (PDF) [file pone.0064491.s003.pdf]

Table S3. Monte Carlo simulations results: ovary- and testis-specific genes in OL gene classes depending on the changes in their flanking genes in the *Drosophila* genus.

Gene order stability definition [1]	Type of genes [2]	Observed [3,4]	Expected (Average ± SD) [3,4]	P_{upper} value [5]	P_{lower} value [5]
OLC	Singleton	2.19	1.19 ± 0.213	0.0001	0.9999
OLC	Unisyntenic	1.81	1.05 ± 0.066	<0.0001	1.0000
OLC	Bisyntenic	0.71	0.95 ± 0.040	1.0000	<0.0001
GO	Singleton	2.00	1.18 ± 0.207	0.0001	0.9999
GO	Unisyntenic	1.67	1.05 ± 0.063	<0.0001	1.0000
GO	Bisyntenic	0.72	0.95 ± 0.042	1.0000	<0.0001
GOO	Singleton	0.90	1.13 ± 0.159	0.9376	0.0626
GOO	Unisyntenic	1.61	1.05 ± 0.061	<0.0001	1.0000
GOO	Bisyntenic	0.74	0.94 ± 0.047	1.0000	<0.0001

1 Gene order stability definitions according to von Grotthuss M, Ashburner M, Ranz JM (2010) *Genome Res*. 20:1084-1096. OLC, overall gene contiguity; GO, gene order; GOO, gene order and orientation.
2 Gene classes defined by the changes in their flanking genes according to von Grotthuss M, Ashburner M, Ranz JM (2010) *Genome Res*. 20:1084-1096. Singleton, each of its flanking genes had changed at least once; unisyntenic, one of its flanking genes had changed at least once, but the other one never changed; bisyntenic, both flanking genes never changed.
3 Original gene expression dataset from Chintapali VR, Wang J, Dow JA (2207) *Nat. Genet.* 39:715-720. Ovary-specific gene, at least one of its probes was deemed as “present” in more than two ovary hybridizations (out of four), and none of its probes was deemed as “present” in more than two testis hybridizations (out of four); testis-specific gene, at least one of its probes was deemed as “present” in more than two testis hybridizations (out of four), and was not deemed as “present” in more than two ovary hybridizations (out of four).
4 Ratio of ovary- and testis-specific genes calculated for observed and expeced by chance distributions of gonadal gene expression tags as: $(n_o / N_o) / (n_t / N_t)$, where n_o and n_t represent the number of ovary- and testis-specific genes in each class of genes, respectively, and, N_o and N_t represent the number of ovary-and testis-specific genes in all three classes of genes, respectively.
5 P_{upper} and P_{lower} values represent the fraction of random simulations with ratios larger or equal, and lower or equal than the observed ones, respectively.
