# Supplementary material for: Females and Males Contribute in Opposite Ways to the Evolution of Gene Order in Drosophila
Source: PLoS One. 2013 May 16;8(5):e64491. doi: 10.1371/journal.pone.0064491 (PMC3655977; doi:10.1371/journal.pone.0064491)
Supplement: Table S4 — Size of Drosophila Lam OLs and D. melanogaster testis-specific gene clusters. (PDF) [file pone.0064491.s004.pdf]

Table S4. Size of *Drosophila* Lam OLs and *D. melanogaster* testis-specific gene clusters.

	**OLC Lam OLs [1,2]**	**GO Lam OLs [1,2]**	**GOO Lam OLs [1,2]**	**Testis-specific clusters [3]**
N	235	241	260	231
Number of genes (Average ± SD)	10.9 ± 8.13	9.9 ± 7.92	7.3 ± 6.34	2.7 ± 1.30
Base pairs (Average ± SD)	162,417 ± 129,184	150,306 ± 126,532	112,089 ± 103,436	19,259 ± 38,008

1 Dataset S1.
2 Gene order stability definitions according to von Grotthuss M, Ashburner M, Ranz JM (2010) *Genome Res*. 20:1084-1096. OLC, overall gene contiguity; GO, gene order; GOO, gene order and orientation.
2 Shevelyov YY, Lavrov SA, Mikhaylova LM, Nurminsky ID, Kulathinal RJ, et al. (2009) Proc. Natl. Acad. Sci. USA 106:3282-3287.
